# Overdiagnosis of a typical carcinoid tumor as an adenocarcinoma of the lung: a case report and review of the literature

**DOI:** 10.1186/1477-7819-10-19

**Published:** 2012-01-23

**Authors:** Ilhan Demirci, Susanne Herold, Andreas Kopp, Michael Flaßhove, Bernd Klosterhalfen, Hermann Janßen

**Affiliations:** 1 Department of General, Visceral, Vascular and Thoracic Surgery, Hospital of Düren; 2 Department of Internal Medicine II, University of Gießen Lung Center, Gießen; 3 Department of Diagnostic and Interventional Radiology, Hospital of Düren; 4 Department of Heamatology and Internal Oncology, Hospital of Düren; 5 The Institute of Pathology, Hospital of Düren

**Keywords:** Overdiagnosis, typical carcinoid tumors, atypical carcinoid tumors, leuprolide, GnrH receptor, Raf-1/MEK/ERK-1/2-pathway

## Abstract

**Background:**

Overdiagnosis of bronchopulmonary carcinoid tumors together with overtreatment can cause serious postoperative consequences for the patient. We report of a patient with a typical bronchopulmonary carcinoid tumor, which was initially misdiagnosed and treated as an adenocarcinoma of the lung. GnrH receptors and the associated Raf-1/MEK/ERK-1/2-pathway are potential targets for analogs in cancer treatment. We suspected a correlation between the lack of tumor growth, application of leuprolide and the Raf-1/MEK/ERK-1/2-pathway. Therefore, we examined GnrH receptor status in the examined specimen.

**Case presentation:**

In 2010 a 77 year-old male patient was shown to have a tumor mass of about 1.7 cm diameter in the inferior lobe of the left lung. Since 2005, this tumor had hitherto been known and showed no progression in size. The patient suffered from prostate cancer 4 years ago and was treated with TUR-P, radiation therapy and the application of leuprolide. We conducted an explorative thoracotomy with atypical segment resection. The first histological diagnosis was a metastasis of prostate cancer with lymphangiosis carcinomatosa. After several immunohistochemical stainings, the diagnosis was changed to adenocarcinoma of the lung. We conducted a re-thoracotomy with lobectomy and systematic lymphadenectomy 12 days later. The tumor stage was pT1 N0 MX G2 L1 V0 R0. Further immunohistochemical studies were performed. We received the results 15 days after the last operation. The diagnosis was ultimately changed to typical carcinoid tumor without any signs of lymphatic vessel invasion. The patient recovered well from surgery, but still suffers from dyspnea and lack of physical performance. Lung function testing revealed no evidence of impairment.

**Conclusion:**

The use of several immunohistochemical markers, careful evaluation of hematoxylin-eosin sections and the Ki-67 labelling index are important tools in discriminating between carcinoids and other bronchopulmonary carcinomas. Although we could not detect GnrH-receptors in the examined specimen, there may be individual differences in expression. GnrH receptor profiles in typical and atypical carcinoids should be scrutinized. This could lead to new therapeutical options, since the GnrH receptor has already been described on atypical carcinoids. Clinically tested drugs such as leuprolide could come to use.

## Background

Laennec was the first one to report on an intrabronchial tumor mass, published posthumously in 1881. This was the first description of a bronchial carcinoid tumor [1]. The first detailed description of a so-called bronchial adenoma, which was probably a carcinoid tumor too, was adduced by Mueller in 1882 [2]. The term "Karzinoid" was introduced by the German pathologist Oberndorfer in 1907 for tumors of the ileum, although Lubarsch had described a similar case of two patients with ileal carcinoid tumors in 1888 [3,4]. Carcinoids represent approximately 2% of all lung tumors. 25-30% of all neuroendocrine tumors appear in the bronchopulmonary system. The age-adjusted incidence rates of bronchopulmonary carcinoids and neuroendocrine tumors in general have increased over the last 30 years for all genders and races. This increase may be due to improvements in histopathological diagnosis and classification and to the more frequent use of endoscopic procedures [5-7]. The average age of diagnosis is at 60 years. Modlin et al. and others described a female predominance [8,9], whereas Quaedvlieg et al. described a female predominance only for patients under the age of 50 years, suggesting hormonal influence [10]. Typical carcinoids are the most common lung tumors in children. Misdiagnosis of typical carcinoid tumors of the lung as SCLC has been reported previously [11,12]. We report of a patient with a typical bronchopulmonary carcinoid tumor, which was initially misdiagnosed as an adenocarcinoma of the lung and also treated as such. This led to serious postoperative consequences for the patient. The tumor described in our case report showed almost no progression in 5 years. GnrH receptors and the associated Raf-1/MEK/ERK-1/2-pathway are potential targets for analogs in cancer treatment. We suspected a correlation between the lack of tumor growth, application of leuprolide and the Raf-1/MEK/ERK-1/2-pathway. Therefore, we examined GnrH receptor status in the examined specimen.

## Case report

**Figure 1:**
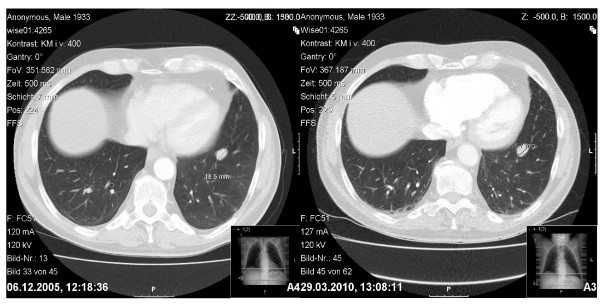
Transversal CT scans of the thorax from the years 2005 (left) and 2010 (right), showing a solid tumor mass in the left inferior lobe of the lung with almost no progress in size. The tumor had a diameter of approximately 1.7 cm in both 2005 and 2010.

**Figure 2:**
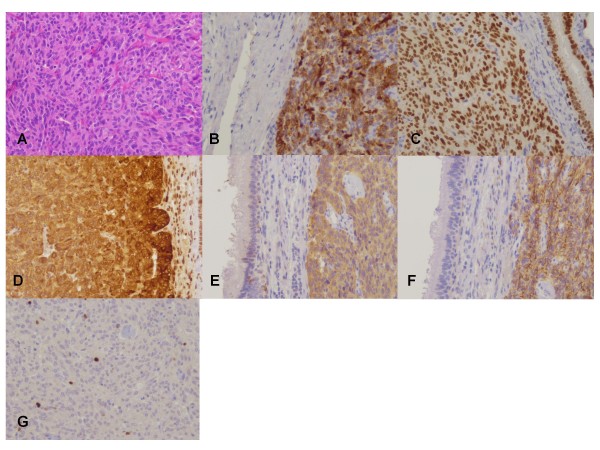
A) HE-staining. B-G) Immunohistochemical stainings. B) Cytoplasmatic expression of CK7. C) Nuclear expression of TTF1. D) Cytoplasmatic expression of chromogranin A. E) Cytoplasmatic expression of synaptophysin. F) Plasma membrane associated expression of CD56. G) Ki-67 labelling index of approximately 5%.

In March 2010 a 77 year-old male patient was shown to have a tumor mass of about 1.7 cm diameter in the inferior lobe of the left lung (see Figure 1). Since 2005, this asymptomatic tumor mass had hitherto been known with a diameter of approximately 1.7 cm. It showed almost no increase in size and could therefore be classified as "stable disease". The patient suffered from prostate cancer 4 years ago and was treated with TUR-P, radiation therapy and the application of leuprolide. Other pre-existing conditions were arterial hypertension and chronic obstructive lung disease. The physical examination was without pathological findings. A preoperative PET/CT scan was not performed due to the fact that neither a positive nor a negative PET/CT scan result rules out a surgical intervention. Therefore, we conducted a left anterolateral thoracotomy with atypical segment resection for curative tumor treatment. The pathological examination of the resected tissue revealed a malignant tumor with a diameter of 1.5 cm. As a first histological diagnosis, a metastasis of prostate cancer with lymphangiosis carcinomatosa was assumed. Further immunohistochemical studies for PSA, CK7, CK20 and TTF1 were performed. Stainings for PSA and CK20 were both negative, stainings for TTF1 showed a strong nuclear and for CK7 a strong cytoplasmatic reaction (see Figure 2). Therefore, the initial diagnosis of a metastasis of prostate cancer was changed to adenocarcinoma of the lung. Relying on this new diagnosis, we conducted a re-thoracotomy with lobectomy and systematic lymphadenectomy as an adequate treatment 12 days after the above-mentioned operation. The resection margins were free of tumor cells. To this point, the tumor stage was pT1 N0 MX G2 L1 V0. Further immunohistochemical studies were performed. These revealed cytoplasmatic expression of chromogranin A and synaptophysin, plasma membrane associated expression of CD56 and a Ki-67 labelling index of approximately 5% (see Figure 2). After re-evaluation of all results and particularly with regard to the little progress in tumor size, the diagnosis was ultimately changed to typical bronchopulmonary carcinoid tumor. A re-examination of the sample showed no invasion of tumor cells in lymphatic vessels. The patient recovered well from surgery and received postoperative rehabilitation therapy. In addition lung function testing revealed no evidence of impairment. Follow-up care is carried out by our department of oncology. The typical carcinoid tumor described in our case report showed almost no progression in 5 years. We suspected a correlation between the lack of tumor growth, application of leuprolide and the Raf-1/MEK/ERK-1/2-pathway. Therefore, we examined GnrH receptor status in the examined specimen. An expression of GnrH receptor could not be detected.

## Discussion

It is generally assumed that carcinoids arise from Kulchitsky cells disseminated in the bronchopulmonary mucosa [13]. Typical carcinoids (TCs) are usually well-differentiated tumors of histologically ordered structure, which are larger than 5 cm with less than 2 mitoses per 10 high power fields and without necrosis. A minority of carcinoids show an atypical appearance with 2-10 mitoses per 10 high power fields, necrosis and they tend to be more aggressive than typical carcinoids. They also show a higher probability to metastasize, to recur and have a worse outcome and prognosis. These are referred to as atypical carcinoids (ACs) [14-16]. The division of neuroendocrine tumors into different subgroups is nowadays based on the WHO classification of 2004 [16]. Other systems like the embryogenetic classification proposed by Wiliams and Sandler in 1963, which differentiated between carcinoids of the foregut (lung, stomach, duodenum, pancreas, upper jejunum), midgut (lower jejunum, ileum, appendix, caecum) and hindgut (colon, rectum), were too inaccurate and therefore could not prevail [17]. 75-90% of all bronchopulmonary carcinoids are localized central, 10-25% peripheral [18,19]. 70-90% are identified as typical and 10-30% as atypical carcinoids [8,20]. TCs are primarily found central and show nodal involvement in 3-20%, whereas ACs are mostly localized in the periphery of the tracheobronchial tree and show nodal involvement in 48-75% [8,21]. ACs are more often associated with smoking than TCs (62-80%). In this regard, a correlation between typical carcinoid histology and smoking is not very likely [6,8,22,23]. The combination of tumor size and mean nuclear area can be used to predict the presence or absence of regional lymph node metastases in 80% and 94% of all cases, respectively [24]. At the time of diagnosis, ACs usually present at a more advanced stage than typical ones. Therefore, an accurate histological diagnosis is essential for further treatment [25]. Mineo et al. published in 2005 that ACs have a higher probability for developing micrometastases, thus explaining the more aggressive clinical behavior. Nodal micrometastases do not correlate with tumor size and stage, implying that even small tumors located in the periphery of the tracheobronchial tree may metastasize. Immunohistochemical detection of micrometastases by using chromoganin A and cytokeratin as markers can help to identify patients with a high risk of recurrence, allowing a more accurate staging [26]. Atypical and typical carcinoid tumors are characterized by a frequent deletion of 11 q material including the MEN1 gene locus. Losses of 10 q and 13 q sequences are more often associated with atypical than with typical carcinoids, making further cytogenetic differentiation possible and probably explaining the more aggressive behavior of ACs [27].

TTF1 is a transcription factor with a molecular weight of 38 kDa, which plays an important role in lung morphogenesis and the expression of surfactant factor. It is considered as a dependable marker in distinguishing between primary and metastatic lung adenocarcinomas and can also be helpful in discerning pulmonary neuroendocrine tumors from gastrointestinal ones. The diagnostic value seems to depend on the used antibody clone [28-30]. According to the literature, the expression rate in adenocarcinomas of the lung varies between 73-92% [31-34]. The described expression rate of TTF1 in bronchopulmonary carcinoids also shows a high variability. Rugge et al. and Sturm et al. were not able to detect TTF1-expression in either TCs or ACs. Furthermore, Sturm et al. showed a positive TTF1-expression in 85.5% and 49% of all studied SCLCs and LCNECs, respectively. According to the authors, this could imply that carcinoids derive from different stem cells than SCLCs and non-SCLCs including LCNECs [35,36]. In this context it is worth mentioning a study published by Sampietro et al., which revealed no relation between carcinoids and small-cell lung carcinomas in gene product immunophenotyping. The authors pointed out that ACs and LCNECs should not be construed as intermediate forms of these two tumor entities [37]. Folpe et al. were able to detect TTF1 in 35% of all cases in TCs, in 100% in ACs, in 75% in LCNECs and in 95% in SCLCs. The authors also concluded that these results suggest a common cell line origin of SCLCs and TCs [38]. Du et al. described that TTF1 is expressed by neuroendocrine cell hyperplasia in 72.7%, by TCs in 27.8%, by ACs in 29.4% and by LCNECs in 37.5% of all cases. They also demonstrated that extrapulmonary neuroendocrine tumors are negative for TTF1, rating it to be 100% specific but not very sensitive for bronchopulmonary carcinoids and possibly also LCNECs [39]. The predominance of TTF1 in carcinoids of bronchopulmonary origin was confirmed by other studies [40-42]. In contrast to these and other publications, Cai et al. gave an account of positive TTF1-expression in 69% of their studied carcinoid cases, all tumors except one were TCs [40]. In addition, immunohistochemical detection of Mib1 and Bcl2 can be useful to differentiate between recurrent and non-recurrent bronchopulmonary carcinoid tumors [35]. The expression of CDX2 and PDX1 is highly specific for identifying neuroendocrine tumors of intestinal origin, since bronchopulmonary carcinoids seem to be negative for these two markers. CK7 is predominantly found in adenocarcinomas of the lung. It is also expressed by 63% of bronchopulmonary carcinoid tumors and by 11% of gastrointestinal carcinoids. CK20 is mainly found in adenocarcinomas of the gastrointestinal tract, but not in bronchopulmonary carcinoid tumors. Together with NESP-55, these mentioned markers can help to discriminate between neuroendocrine tumors or adenocarcinomas of pulmonary and such of extrapulmonary origin [34,40-43]. Chromoganin A (CgA) and synaptophysin are commonly used neuroendocrine markers in immunohistochemical analysis [44]. Overdiagnosis of typical carcinoid tumors of the lung as SCLCs has been reported previously [11,12]. In our case report, the first diagnosis was metastasis of prostate cancer. After initial immunohistochemical stainings and due to the therein reported expression of TTF1, CK7 and the lack of CK20, this diagnosis was changed to primary adenocarcinoma of the lung. Further immunohistochemical stainings were performed. In consideration of the slow tumor progression, the low Ki-67 labelling index and the expression of CgA and synaptophysin, the diagnosis was ultimately changed to TC. Up to this time, we had already conducted a lobectomy to treat the previously suspected adenocarcinoma. As Pelosi et al. stated, careful evaluation of hematoxylin and eosin sections, together with the Ki-67 labelling index, remains the most important tool in discriminating between carcinoids and SCLCs. An index below 20% is typical for carcinoid tumors, whereas an index above 50% is usually found in SCLCs.

GnrH receptors and the associated Raf-1/MEK/ERK-1/2-pathway are potential targets for analogs in cancer treatment. Activation of the Raf-1/MEK/ERK-1/2-pathway by leflunomide and teriflunomide can inhibit proliferation and growth of BON carcinoid cells in vitro and in vivo and also decrease expression of neuroendocrine markers in both BON and H727 carcinoid cells [45]. Similar effects have been reported for estradiol, ZM336372 and tautomycin [46-50]. Kidd et al. detected GnrH receptor expression in H720, but not in H727 cells and GHRH receptor expression in H727, but not in H720 cells. According to their results for H720 cells, GnrH+doxorubicin conjugate is a more effective proliferation inhibitor in vitro than doxorubicin alone. In H727 cells, this difference was not observed. Furthermore, GHRH receptor antagonists have an antiproliferative effect in H727 but not in H720 cells [51]. The signaling mechanisms of GnrH receptors differ significantly from cell type to cell type. For example, signaling in COS7 cells is not dependent on PKC. Since GnrH receptor expression has not been studied in larger patient collectives with carcinoid tumor disease, this raises the question whether there is an association with the above mentioned Raf-1/MEK/ERK-1/2-pathway and if its activation is dependent on GnrH receptors [52]. If there is such a context, the effects of agonists like leuprolide as a GnrH receptor agonists may become of interest. Although we could not detect GnrH-receptors in the examined specimen, there may be individual differences in expression. In this regard, GnrH receptor profiles in TCs and ACs should be scrutinized. Further investigations could lead to new therapeutical options in the treatment of TCs and especially ACs, since the GnrH receptor has been described as located on the latter [51]. Clinically tested and approved drugs such as leuprolide could come to use.

According to an analysis of eight studies by Gustafsson et al., approximately 58% of all patients show unspecific symptoms: cough in 32%, hemoptysis in 26%, pneumonia in 24% of all cases and, to a lesser extent, fever, dyspnoe and chest pain. A carcinoid syndrome occurs seldomly in approximately 1-3% [53]. Long term obstruction typical for centrally localized tumors can cause multiple bronchiectases in 19% of all patients [22]. Young and adult patients should undergo further examination if they show long-standing pulmonary symptoms without any response to drug therapy [54,55]. To date there is no laboratory test available that could always detect a carcinoid reliably. Several serum markers are used. CgA is a maker of carcinoids in general with a high sensitivity (85.7%) and specificity (67.9%), although it can also be detected in SCLCs depending on the clinical stage [56,57]. The elevation of CgA can indicate the recurrence of radically operated midgut carcinoid tumors in 85% of all cases. The measurement of CgA can be recommended for the continuous surveillance of carcinoid patients [57,58]. However, CgA is also known to be false positive in some cases, for example in patients with multiple myeloma, renal impairment, atrophic gastritis or proton pump inhibitor therapy [59]. The measurement of 5-HIAA and, according to the clinical symptoms, of ACTH, MSH, GH etc. is only useful if a carcinoid syndrome is suspected. Various diagnostic methods are used in combination. Only 75% of all patients show a suspicious chest X-ray. Despite a lack of sensitivity and specificity, high resolution CT scans of the abdomen and thorax are used most commonly for an initial localization of the tumor. Carcinoid tumors are known to be highly vascularized and may show a strong contrast agent enhancement. MRI can provide additional information. ^68^Ga-DOTA-peptide PET/CT scans are currently the most accurate imaging procedures for the diagnosis of NETs such as bronchial carcinoids with a sensitivity of 97% and a specificity of 92%. Even the detection of tumors smaller than 1 cm is possible. According to a study published by Ambrosini et al., ^68^Ga-DOTANOC PET/CT either affected stage or caused a therapy modification in more than 50% of the NET patients, proving its superiority to conventional imaging [60]. In this context it should be noted that it can be distinguished between endobronchial carcinoid and inflammatory myofibroblastic tumors by combining ^18^F-FDG PET and ^68^Ga-DOTATOC PET/CT [61]. Prior to this new method, somatostatin receptor scintigraphy was used in imaging, which was not very specific for bronchial carcinoids. A recently published study showed that ^68^Ga-DOTANOC PET/CT is superior to ^111^In-pentetreotide in the detection of CUP-NETs, with a localization rate of the primary tumor of 59% vs. 39%, respectively. The authors concluded that this method can play a major role in the management of patients with CUP-NETs [62]. Bronchial carcinoids are known to be highly vascularized. Nevertheless they show a high rate of false negative results in ^18^F-FDG PET, although solitary cases of carcinoids with high ^18^F-FDG uptake have been reported [63-65]. Interestingly, combined diagnostic imaging with ^131^I-MIBG and ^111^In-pentetreotide achieved an overall sensitivity of 95% for localization of carcinoid tumors. However, this approach was not further pursued [66]. According to the current ACCP guidelines, transthoracic needle biopsy, bronchoscopy or even surgical resection are appropriate means of diagnosis for solitary pulmonary nodules of at least 8-10 mm diameter. VATS, thoracotomy and mediastinoscopy can be used in combination or as sole diagnostic procedures [67]. Endo- and epibronchial ultrasound can also provide further valuable information [68,69]. In this regard, it is important to mention that transthoracic fine needle aspiration biopsy bears the risk of nondiagnostic biopsies. It is dependant on many different factors like tumor size or needle diameter [67,70]. Also, there are several reports on implantation metastasis caused by fine needle aspiration biopsy. Although FNAB is considered to be a well-established diagnostic procedure, we think the above mentioned limitations and complication threaten the success of any curative therapy and should not be ignored [71]. ^18^F-FDG PET/CT scan is known to be highly sensitive, yet has its limitations due to a lower specifity. Like the above mentioned FNAB, a negative PET/CT scan does not rule out the possiblity of malignancy of a solitary pulmonary nodule and cannot replace surgical resection [70]. Therefore, we did not see the necessity to perform a preoperative PET/CT scan. Since surgical resection is still considered to be the most reliable diagnostic test [67,70], we prefer surgical diagnostic procedures, if they are feasible. Histological analysis with further immunohistochemical differentiation still remains the gold standard in diagnosing bronchopulmonary carcinoid tumors, with markers like TTF1, CK7, CK20, chromogranin A, synaptophysin and Ki-67 being used routinely.

Surgical resection is still considered to be gold standard in treatment. Nowadays, tissue-sparing procedures become more and more popular, since they show no great difference in long-term survival and a better postoperative quality of life. For this purpose, early diagnosis is essential. The extents of surgical resection and lymph node dissection are still a matter of ongoing debate. Some authors concluded that parenchyma sparing procedures in ACs should be avoided because of a higher recurrence rate [25,72] and are suitable especially for TCs. Others prefer more invasive procedures such as lobectomy in combination with radical lymphadenectomy for any histological kind of carcinoid tumor [20,22]. Endobronchial typical carcinoids with strictly endoluminal growth and small tumor base are reported to be treatable by bronchoscopic resection, showing excellent long term results [69]. Wedge bronchoplasty with large margins should be avoided due to the risk of kinking of the tracheobronchial tree [73]. Some authors pointed out that lymph node dissection should be performed in any patients with atypical carcinoids [74,75], whereas others recommended systematic lymphadenectomy in any carcinoid case [20,22,24,75]. Octreotide and chemotherapy are normally used if a carcinoid syndrome or metastases are present. Kaplan et al. even recommended postoperative radiochemotherapy for patients with atypical carcinoids at stage I, reasoning it with higher locoregional failure and distant metastasis rates as compared to patients with typical carcinoids [76]. According to the ACCP guidelines, there is level 2 C evidence for a conservative and observant approach in case of a new diagnosed pulmonary tumor mass without growth in the last 2 years. However, this strategy is not used very often in practice, because tumors greater than 1.5 cm in diameter show high potential for malignancy [67]. This approach should therefore be used with caution. Even after radical resection, distant metastases can occur in 8% of all TC cases and are associated with a worsening of prognosis.

The 10-year survival rate for typical carcinoids ranges from 82% to 93%, for atypical carcinoids from 56% to 64% [8,69,72,75]. Modlin et al. described a 5-year survival rate of 73,5% for all stages [6]. The various forms of surgical resection techniques used nowadays normally show excellent long-term results. Histology, evidence of invasive growth, nodal status and the presence or absence of distant metastases were identified as the most important prognostic factors for long term survival [6,7,26,72,75]. At the time of diagnosis, ACs usually show a more advanced stage than TCs. According to a recently published study, nodal invasion in TCs has no influence on prognosis [9]. In contrast, Martini et al. reported in 1994 that long term survival is independent of lymph node metastases for both TCs and ACs and that recurrence seems to depend more on cell type than nodal status. However, this study only consisted of 25 patients [77]. In case of metastatic disease, TCs may show a similar clinical course like ACs. If the metastases are not resectable, the disease is considered to be incurable [53,78,79].

## Conclusion

Misdiagnosis of bronchopulmonary carcinoid tumors with surgical overtreatment can cause serious consequences for the patient like intra- and postoperative complications with further possible serious limitations of lung function. The use of several immunohistochemical markers and careful evaluation of hematoxylin and eosin sections together with the Ki-67 labelling index are important tools in discriminating between carcinoids and other bronchopulmonary carcinomas. Simultaneously, the pre-operative progression of the disease should be considered before defining the extent of initial surgical resection. In case of metastatic disease, TCs may show a similar clinical course like ACs and a significant worsening of prognosis. Therefore, we think that even small TCs which are considered as "stable disease" like our reported case should be resected. Also, a 10-year follow up is recommended.

GnrH receptors and the associated Raf-1/MEK/ERK-1/2-pathway are potential targets for analogs in cancer treatment. Activation of the above mentioned pathway can inhibit proliferation and growth of various carcinoid cell types. The typical carcinoid tumor described in our case report showed almost no progression in 5 years. The patient suffered from prostate cancer 4 years ago and was treated with TUR-P, radiation therapy and the application of leuprolide. We suspected a correlation between the lack of tumor growth, application of leuprolide and the Raf-1/MEK/ERK-1/2-pathway. Therefore, we examined GnrH receptor status in the examined specimen. Although we could not detect GnrH-receptors in the examined specimen, there may be individual differences in expression. In this regard, GnrH receptor profiles in TCs and ACs should be scrutinized. Further investigations could lead to new therapeutical options in the treatment of TCs and especially ACs, since the GnrH receptor has already been described on the latter. Clinically tested and approved drugs such as leuprolide could come to use.

## Consent

Written informed consent was obtained from the patient for publication of this Case report and any accompanying images. A copy of the written consent is available for review by the Editor-in-Chief of this journal.

## List of abbreviations

TCs: typical carcinoid tumors; ACs: atypical carcinoid tumors; CgA: Chromogranin A; SCLC: small cell lung carcinoma; LCNECs: large cell neuroendocrine carcinomas.

## Competing interests

The authors declare that they have no competing interests.

## Authors' contributions

ID: preparation and drafting of the manuscript, literature research; SH: editing of the manuscript; AK: editing of the manuscript; BK: immunohistochemical staining and examination of the specimen, editing of the manuscript; MF: editing of the manuscript; HJ: editing of the manuscript. All authors read and approved the final manuscript.
